# Harnessing the potential of recombinant human BMP2: regulating scleral changes in myopic guinea pigs

**DOI:** 10.3389/fmed.2025.1526656

**Published:** 2025-05-01

**Authors:** Yijie Liu, Qi Hao, Xuemei Pan, Pubo Wang, Dadong Guo, Qingmei Tian, Xiuyan Zhang, Xiuzhen Lu, Qiuxin Wu, Hongsheng Bi

**Affiliations:** ^1^Shandong University of Traditional Chinese Medicine, Jinan, Shandong, China; ^2^Shandong Key Laboratory of Integrated Traditional Chinese and Western Medicine for Prevention and Therapy of Ocular Diseases, Shandong Academy of Eye Disease Prevention and Therapy, Affiliated Eye Hospital of Shandong University of Traditional Chinese Medicine, Jinan, Shandong, China

**Keywords:** myopia, recombinant human bone morphogenetic protein 2, BMP/smad, scleral remodeling, scleral

## Abstract

Studies have shown that the development of myopia is associated with scleral remodeling, but the exact mechanism is not yet clear. This study investigates the effects of vitreous injection of recombinant human bone morphogenetic protein 2 (rhBMP2) on scleral remodeling in myopic guinea pigs and the possible signaling pathways. Guinea pigs were randomly divided into normal control (NC) group, lens-induced myopia (LIM) group, rhBMP2 low-dose group (LD), rhBMP2 medium-dose group (MD), and rhBMP2 high-dose group (HD). After rhBMP2 intervention, myopic refraction was reduced and axial growth was delayed compared with the LIM group; Hematoxylin–eosin (H&E) staining showed that the arrangement of scleral collagen fibers was loose, the disorder was improved, and the cavities were reduced, especially in MD group; and immunofluorescence staining showed elevated *α*-SMA protein expression. Q-PCR and western blot results showed that after rhBMP2 intervention, at the mRNA and protein levels, the expression of BMPRIA, smad1, smad5, smad9, smad4, TIMP2, and Col1A1 was up-regulated, and MMP2 expression was down-regulated when compared with the LIM group. From this study, we conclude that after injecting rhBMP2 into the vitreous cavity of experimental myopic guinea pigs, it can bind to BMP2-related receptors, activate smad signaling pathway, affect the expression of MMP2/TIMP2, promote the expression of Col1A1 gene, regulate scleral remodeling, promote collagen I synthesis, and delay the development of myopia.

## 1 Introduction

In recent decades, myopia has become a worldwide public health problem. According to statistics, nearly 28.3% of the world’s population suffers from myopia which shows a sharp rising trend. It is expected that by 2050, the total number of myopia will account for half of the global population (1). Myopia will not only affect the visual quality of patients, high myopia can cause serious complications, such as retinal detachment, choroidal neovascularization, glaucoma, and so on, which will lead to serious visual loss and irreversible damage.

Myopia mainly occurs after birth during the growth stage of the eyeball. High myopia is characterized by axial elongation of the eye and thinning of the posterior sclera. Scleral tissue is composed of two parts, a small part of the cellular component and the majority of the extracellular matrix. The remodeling of the sclera in myopia is generally considered to be the change of the scleral extracellular matrix, and collagen is the main component of the extracellular matrix. Studies have shown that the expression of collagen in myopic scleral remodeling is all decreased, with the most significant reduction in the expression of type I collagen fibers (2, 3).

Bone morphogenetic proteins (BMP) are polypeptide growth factors produced by osteoblasts, all of which are transforming growth factor-*β* (TGF-β) except BMP1. A member of the TGF-beta superfamily. BMP receptors belong to the receptor superfamily and are composed of typeI and typeII subtypes. BMP receptors (BMPR-IA, BMPR-IB, BMPR-II) were found to be expressed in human and guinea pig sclera (4–6). BMP activates the Smad signaling pathway system by binding to BMP receptors on the cell membrane, and further activates or inhibits the expression of related genes in the nucleus, thus affecting cell growth, differentiation, and extracellular matrix synthesis (7).

BMP2 has a regulatory effect on scleral remodeling by participating in scleral remodeling to regulate eye axis elongation. It was found that the expression of BMP2 was downregulated in animal models of myopia. Li et al. (8) in −4D LIM guinea pigs, by lens-induced myopia, the expression of BMP2 in the sclera of the posterior pole decreased after 3 weeks, but recovered in 1 week after removing the −4D lens, with no difference compared with the control eye. Zhang et al. (9) conducted form-deprivation myopia (FDM) experiments on C57BL/6 mice and found that the expression of BMP2 was significantly down-regulated and the morphological characteristics of the sclera were changed. In addition, *in vitro* cell experiments confirmed that BMP2 can promote scleral cell proliferation and regulate scleral remodeling (8).

The above studies fully demonstrated that BMP2 is closely related to scleral remodeling during myopia. Based on the above points, we injected rhBMP2 into the vitreous cavity of LIM guinea pigs to observe the changes in refraction and axial length of the eye, as well as the changes in the expression of related mRNA and protein in the sclera, to further explore the influence of rhBMP2 on scleral remodeling and the possible signaling pathway, providing a theoretical basis for the prevention and treatment of myopia.

## 2 Materials and methods

### 2.1 Animals

The present study was approved by the Ethics Committee of Shandong University of Traditional Chinese Medicine and adhered to the ARVO Statement for the Use of Animals in Ophthalmic and Vision Research. Before enrollment, guinea pigs with various ocular conditions, including cataracts and corneal disease, were excluded. In the present study, 2-week-old male guinea pigs (*Cavia porcellus*, English short-hair stock, tricolor strain; Danyang Changyi Experimental Animal Company, Jiangsu, China) with a mean weight between 100 and 120 g were chosen for the relevant experiments. The guinea pigs were raised at a constant temperature of 25°C ± 2°C with humidity ranging from 40% to 70%; noise levels were less than 60 dB. The experiments were conducted with natural light during the daytime, with a 12/12-h light/dark cycle and a light intensity of 500 lux in the cage.

### 2.2 Myopic model building and grouping

The qualified guinea pigs (90 animals) were divided into the following five groups of 18 animals, each using a random number table: the normal control (NC) group, the lens-induced myopia (LIM) group, the low dose (LD) group, the medium dose (MD) group, and the high dose (HD) group. The right eye of guinea pigs in LIM group, LD group, MD group, and HD group was covered with −6.0 D lens to induce myopia, and the left eye was used as self-control, while both eyes of guinea pigs in NC group were not treated. To ensure the validity of the LIM model in guinea pigs, all lenses were cleaned and wiped every morning and evening and replaced promptly if there were obvious scratches. Guinea pigs in the LIM group were injected with 4 μL of linger injection solution in the vitreous cavity of the right eye. The guinea pigs in LD, MD, and HD groups were injected with rhBMP2 (25 μg/mL) 1 μL, 4 μL, 8 μL intravitreal cavity in right eye, once every 3 days.

### 2.3 Strip photometry

The guinea pigs in each group were subjected to streaking retinoscope before inclusion and 2w and 4w after modeling. Before the examination, 10 mg/mL of cyclopentolate hydrochloride eye drops (Alcon, Geneva, Switzerland) were dropped into the conjunctival capsule of the guinea pigs three times, one drop each time, at an interval of 5 min. Refractive examinations were performed 30 to 45 min after the last eye drop application. The working distance was 50 cm, the whole process was operated by the same skilled optometrist in the dark room, and the refraction was the average of the vertical and horizontal meridian tests. Each eye was measured at least six times, and the average value was taken as the experiment result.

### 2.4 Axial length

The axial length was measured after 0, 2, and 4 weeks of modeling. Ophthalmic A-type ultrasonography (Cinescan, Quantel Medical, Cournon-Auvergne, France) was used for the measurement of ocular axis length. The eye surface was anesthetized with 1% oxybuprocaine hydrochloride eye drops (Benoxicam, Japan) before the A-type ultrasonography measurement. Then, the probe is placed perpendicular to the anterior apex of the cornea and measurements are taken continuously. The anterior chamber propagation velocity was 1,557 m/s, the lens propagation velocity was 1,723 m/s, and the vitreous propagation velocity was 1,540 m/s. A total of 10 readings were averaged to serve as the final one, as described previously. Continuous measurement 10 times, eliminate the obvious deviation of the value, and take the average, the whole process is completed by the same technician.

### 2.5 Intravitreal injection

Routine use of antibiotic eye drops in the right eye a day in advance, disinfection of surgical instruments, and a 6-h ban on animal food and water. Before the operation, 10 mg/mL of cyclopentolate hydrochloride eye drops (Alcon, Geneva, Switzerland) were dropped into the conjunctival capsule of the guinea pigs twice, one drop at a time. Observe that the pupil diameter of the animal is enlarged to 3 mm. Animals were gas anesthetized with isoflurane and operated inside a sterile workbench. Under the microscope, the eyeball was fixed with microscope tweezers, and the microsyringe was inserted into the sclera vertically 1 mm behind the temporal limbal of the cornea. After the breakthrough, the needle was tilted 20° toward the ocular wall to avoid the lens, and the rhBMP2 was injected by advancing about 3 mm. After the injection, the needle hole was gently held with tweezers for 30 s. The eye was coated with ofloxacin ointment, covered with sterile gauze, and wrapped with medical tape. Control room temperature to about 25°C to keep warm, continue to wear glasses after surgery to induce myopia.

### 2.6 Animal materials

After 2 and 4 weeks of modeling, the guinea pigs in each group were anesthetized by intraperitoneal overinjection of 40 g·L^−1^ pentobarbital, and the right eyeball was removed immediately after death, rinsed in 0.9% sodium chloride solution at 4°C. Next (Ice operation), the conjunctiva, fascia, and extraocular muscles attached to the outside of the guinea pigs’ eyeball were removed under the microscope, removed the optic nerve and the eyeball were cut open at the junction of the cornea and sclera. Remove the vitreous, the retina and choroid are gently scraped out. Scleral tissue was frozen in liquid nitrogen and stored at −80°C for later use.

### 2.7 Histopathological staining

After 4 weeks of modeling, 40 g·L^−1^ pentobarbital was injected intraperitoneally, and the guinea pig eyeballs were removed, rinsed in sterile normal saline, and periocular tissue was removed, immediately placed in eyeball fixation solution for 24 h, and then routine dehydration, paraffin embedding, and section were performed. Hematoxylin–eosin (H&E) staining was performed to observe the physiological morphological and structural changes of the scleral of each guinea pig’s model eye. Three guinea pigs were used in each group.

### 2.8 Immunofluorescent microscopy

After 4 weeks of modeling, guinea pigs were anesthetized by intraperitoneal injection of 40 g·L^−1^ pentobarbital. Three guinea pigs were used in each group. The eyeballs of guinea pigs were removed, washed with sterile normal saline, excess connective tissue, and extraocular muscle were removed, and frozen sections were made after embedding in OCT gel. Sections were washed three times with PBS, and 5% BSA sealing solution was added dropwise and incubated at 37°C for 30 min. The *α*-SMA antibody (dilution 1:500; Bioss, Beijing, China) was added dropwise, and the reaction was fully reacted at 4°C overnight. After rewarming for 45 min, the sample was washed three times with PBS, and the secondary antibody (dilution 1:10,000; Bioss, Beijing, China) was added dropwise, incubated for 30 min at 37°C, and washed three times with PBS under the condition of light protection. After sealing the film with a drop of DAPI-containing antifluorescent attenuator, it was observed by Zeiss laser scanning confocal microscope LSM780.

### 2.9 Real-time fluorescent quantitative PCR

Scleral tissues from 4-week-old guinea pigs were extracted and frozen in liquid nitrogen. Six guinea pigs were used in each group. Then, equal amounts of scleral tissues were taken and ground separately using a modified tissue/cellular RNA rapid extraction kit (SparkJade Science Co., Ltd., Jinan, China) to extract total RNA. The RNA purity and concentration were measured by an ultraviolet spectrophotometer (K5600; Beijing Kaiao Technology Development Co., Ltd., Beijing, China), the RNA 260/280 ranged from 1.8 to 2.0, and the concentration was greater than 100 ng/μl. The HiScript II Q RT SuperMix for qPCR (+gDNA wiper) (Vazyme Biotech Co., Ltd., Nanjing, China) was used for reverse transcription to obtain the cDNA of the target genes. Real-time quantitative PCR (qPCR) was performed using a 96-well plate (NEST Biotechnology, Wuxi, China) to detect the levels of BMPRIA, BMPRIB, BMPRII, smad1, smad5, smad9, smad4, tissue inhibitor of metalloproteinase 2 (TIMP2), matrix metalloproteinase 2 (MMP2), collagen I and aggrecan levels. The primer sequences are listed in Table 1. The qPCR conditions were as follows: Pre-incubation 95°C for 3 min; Amplification 95°C for 20 s, 60°Cfor 15 s for 45 cycles; melting curves 95°C for 5 s, 65°C for 1 min, and 97°C continuous. The expression level of the target gene in each sample was normalized to the internal reference level of glyceraldehyde 3-phosphate dehydrogenase (GAPDH), and the result was analyzed using the 2^−△△ CT^ method.

**Table 1:** Primer sequence of the target genes.

Gene		Primer Sequences (5′-3′)
GAPDH	Forward	TCAAGAAGGTGGTGAAGCAGG
	Reverse	CTGTTGCTGTAGCCGAACTCAT
BMPRIA	Forward	GGAGCAGAGAACAAGACGACGAAC
	Reverse	CAAGTGATTCCACCGCAGCAGAG
BMPRIB	Forward	CAGTACCAGCCTTGAACACCATCC
	Reverse	ACCCAATGTCTCCCTCCTCCAAC
BMPRII	Forward	CTGATGCACTGTGGAGAGGTTGTC
	Reverse	TGTGAGAAGGTGGCAGAAGGAGAG
Smad1	Forward	GGCTGCCTCACGTCATCTACT
	Reverse	AACAGGGCTTTCTACTCGCTTAT
Smad5	Forward	TAGTCCAGCAGTCAAGCGATTGTTG
	Reverse	GCTCAAGGCTTTCTCCAGTTCCTC
Smad4	Forward	CCACAGGACAGAAGCCATTGAGAG
	Reverse	CACTAAGGCACCTGACCCAAACATC
Smad9	Forward	GTGGAGAAGTGTATGCCGAGTGC
	Reverse	AGAGCTGGTTGTTGAAGACCTTGAG
MMP2	Forward	GATGCCTTTGCTCGAGCCTT
	Reverse	GCCCATCCTTCCCATCGAAC
TIMP2	Forward	TGTCGGTGGGAAGAAGGAATATC
	Reverse	TCTGGGTGGTACTCAGGGTGTC
Aggrecan	Forward	AGCAGCAGAGGAGAGCACAGAG
	Reverse	CACTGGGCATTGCAGGAGAAGG
Col1a1	Forward	CAAGTTGTGGACCAAGACAGAGAC
	Reverse	CCTTACAGGTCATCTCTTCGCAC

### 2.10 Western blot analysis

To investigate the protein levels of the related molecules, we used western blot assays to determine protein levels of guinea pigs in each group. Six guinea pigs were used in each group. After 4 weeks of myopia induction, were randomly selected in each group, and the scleral tissues were isolated. The sclera tissue was ground with liquid nitrogen, and add 60 μL of tissue lysate for every 20 mg of issue, after ultrasonic crushing, the sclera tissue was cracked overnight at 4°C.

The supernatant was collected and stored at −20°C after centrifugation at 12,000 rpm for 15 min at 4°C. The protein concentration was measured by BCA method, and the sample was diluted appropriately according to the protein concentration. In the present study, 10% sodium dodecyl sulfate–polyacrylamide gel electrophoresis (SDS–PAGE) was used to separate the target proteins, and a polyvinylidene difluoride (PVDF) membrane was used for membrane transfer. An appropriate amount of concentrated SDS-PAGE protein loading buffer was added to the collected protein samples. Heat 100°C or boiling water for 10 min to fully denatured proteins. Configuration of 10% separation glue and 5% concentrated glue, the loading amount of protein per well was 25 μg, electrophoresis (70 V, 30 min) after bromophenol blue reaches the boundary between laminated glue and separator glue, the electrophoresis continued (110 V, 90 min) and ended. The membrane was transferred at a contrast current of 200 mA. After the membrane transfer, the PVDF membrane was placed in 5% skim milk solution (skim milk powder 1 mg: 20 mL TBST) and enclosed in a shaker at room temperature for 1.5 h. Then, washing with TBST twice, 5 min each time. PVDF membrane was put into a clean homemade closed bag, with the diluent of primary antibody added, and placed in a shaker at 4°C overnight for incubation. PVDF membrane was cleaned with TBST for 4 times, 8 min each time, second antibody diluent was added, and incubated at room temperature for 1 h on the shaking bed. After incubation of the second antibody, TBST was used for 4 times, 8 min each time. TBS cleaning twice, 5 min each time; After mixing the developing solution AB in equal volume, cover the film for color development for 2 min. FX multifunctional imaging system is applied to develop and fix images immediately after 10 to 30 s of exposure.

Finally, we used the FUSION-FX7 imaging system (Vilber Lourmat, Marne-la-Vallée, France) for development using DAB (Sigma-Aldrich, St. Louis, MO, United States) and quantified results using fusion CAPT software (Vilber Lourmat).

### 2.11 Statistical analysis

All experiments were performed in triplicate and the results were expressed as mean ± standard deviation (Mean ± SD). SPSS Statistics 23.0 (IBM, Chicago, IL, United States) was used for statistical analysis. One-way ANOVA was used for overall comparison of data between groups, and LSD-t test was used for intergroup comparison between groups. *p* < 0.05 was considered statistically significant.

## 3 Results

### 3.1 The change of spherical equivalent refraction

There was no significant difference in spherical equivalent refraction (SER) among all groups at week 0 before modeling (Table 2). After 2 and 4 weeks of modeling, the myopia refraction of LD, MD, and HD groups were lower than that of LIM group, and the myopia refraction of MD group was the lowest. These results indicate that vitreous cavity injection of rhBMP2 can delay the increase of myopic refraction in LIM guinea pigs, especially in the medium-dose group.

**Table 2:** SER after cycloplegia in each group (mean ± SD, D).

	0w	2w	4w
NC	1.37 ± 0.85	−1.27 ± 1.49	−2.06 ± 1.41
LIM	1.42 ± 0.93	−4.05 ± 1.22^#^	−6.01 ± 0.99^#^
LD	1.40 ± 0.61	−3.23 ± 1.31^*^	−4.20 ± 1.34^*^
MD	1.40 ± 0.71	−1.77 ± 1.14^*^	−3.49 ± 0.86^*^
HD	1.42 ± 1.14	−2.81 ± 0.96^*^	−4.01 ± 0.87^*^
*F* value	0.24	32.448	73.190
*p* value	0.999	<0.001	<0.001

^#^*p* < 0.05, Compared with NC group; ^*^p < 0.05, Compared with LIM group. NC, Normal control; LIM, Lens-induced myopia; LD, low dose; MD, medium dose; HD, high dose (*n* = 90).

### 3.2 Changes of axial length

**Figure 1:**
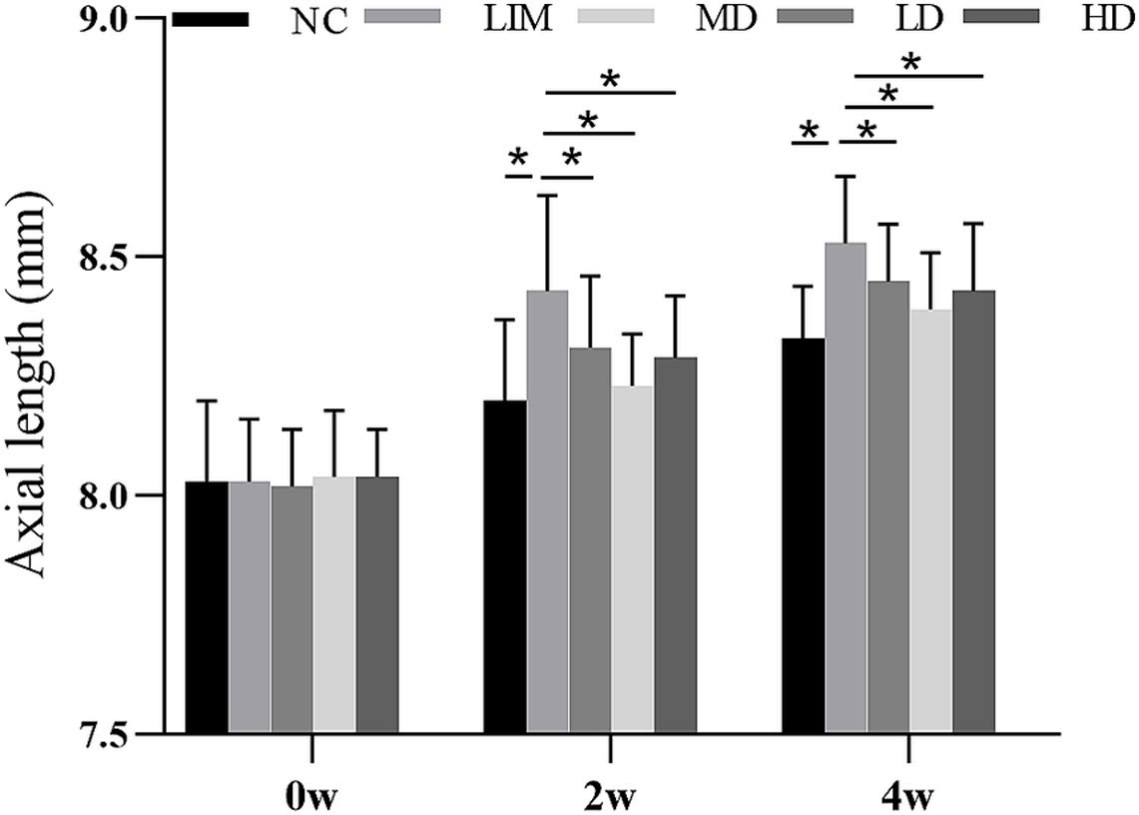
Changes of axial length before and after molding (mean ± SD, mm). NC, Normal control; LIM, Lens-induced myopia; LD, low dose; MD, medium dose; HD, high dose (*n* = 90) * *P* < 0.05.

As can be seen from Figure 1, there was no significant difference in axial length (AL) among all groups at week 0 before modeling. After 2 and 4 weeks of modeling, the AL of eyes in LD, MD, and HD groups was shorter than that in LIM group, and the AL of eyes in MD group was the shortest. These results indicate that vitreous cavity injection of rhBMP2 can delay the AL growth of experimental myopic guinea pigs, especially in the medium-dose group.

### 3.3 H&E staining analysis

**Figure 2:**
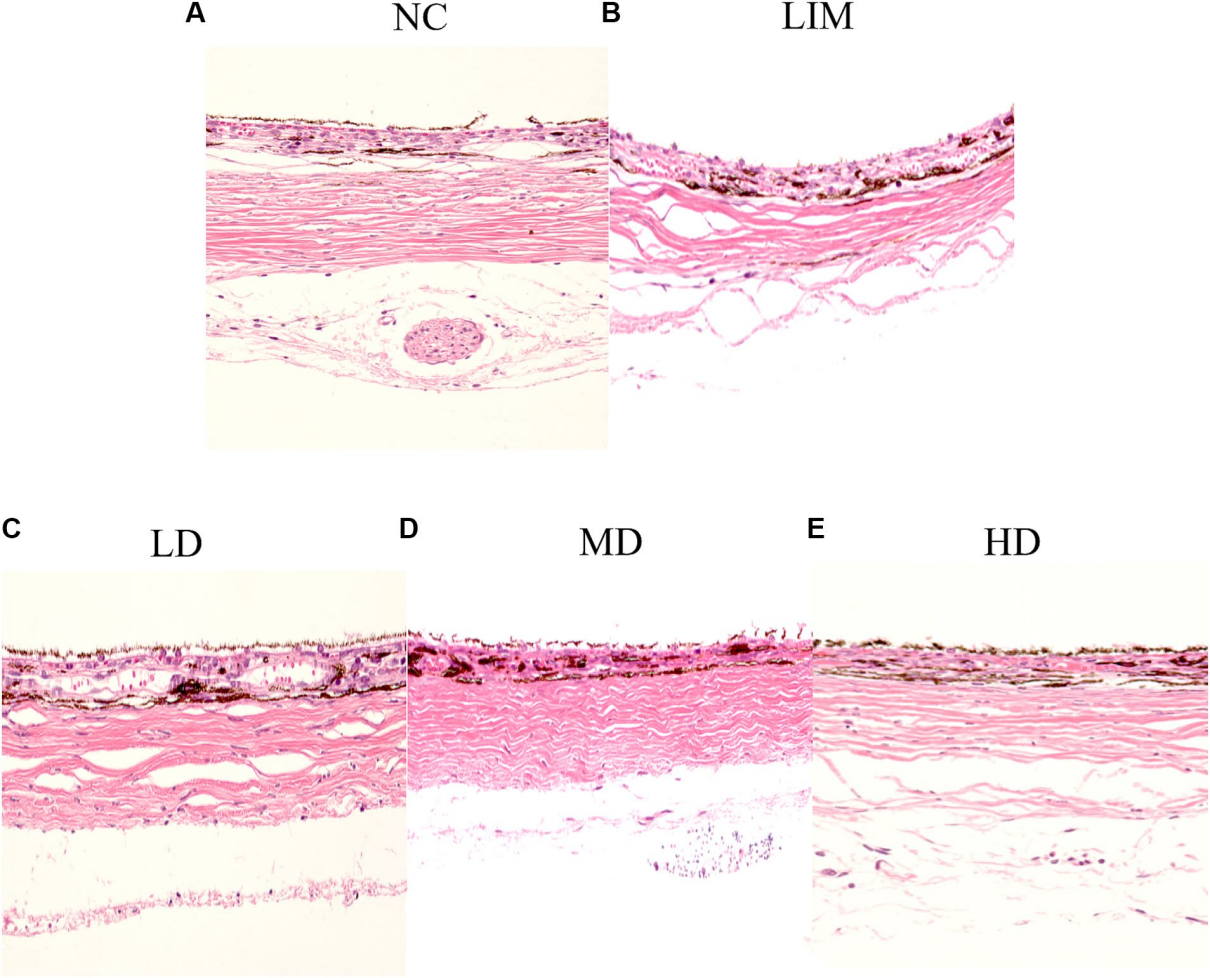
After 4 weeks of modeling, HE staining was performed to observe the scleral structure changes, 200×. **(A)** Normal group, **(B)** LIM group, **(C)** Low-dose group, **(D)** medium-dose group, **(E)** High-dose group (*n* = 3).

As shown in Figure 2, the scleral structure changed 4 weeks after the molding. Compared with the normal group, the sclera in the posterior pole of the LIM group showed scleral thinning, collagen fiber thinning, loose and disorganized arrangement, and obvious lacunae, suggesting remodeling of the scleral extracellular matrix after the formation of myopia. Compared with LIM group, the arrangement of scleral collagen fibers in LD, MD, and HD groups was loose, the disorder was improved, and the space was reduced, especially in medium-dose group. These results suggest that intravitreal injection of rhBMP2 can delay scleral extracellular matrix remodeling.

### 3.4 Immunofluorescence staining analysis

**Figure 3:**
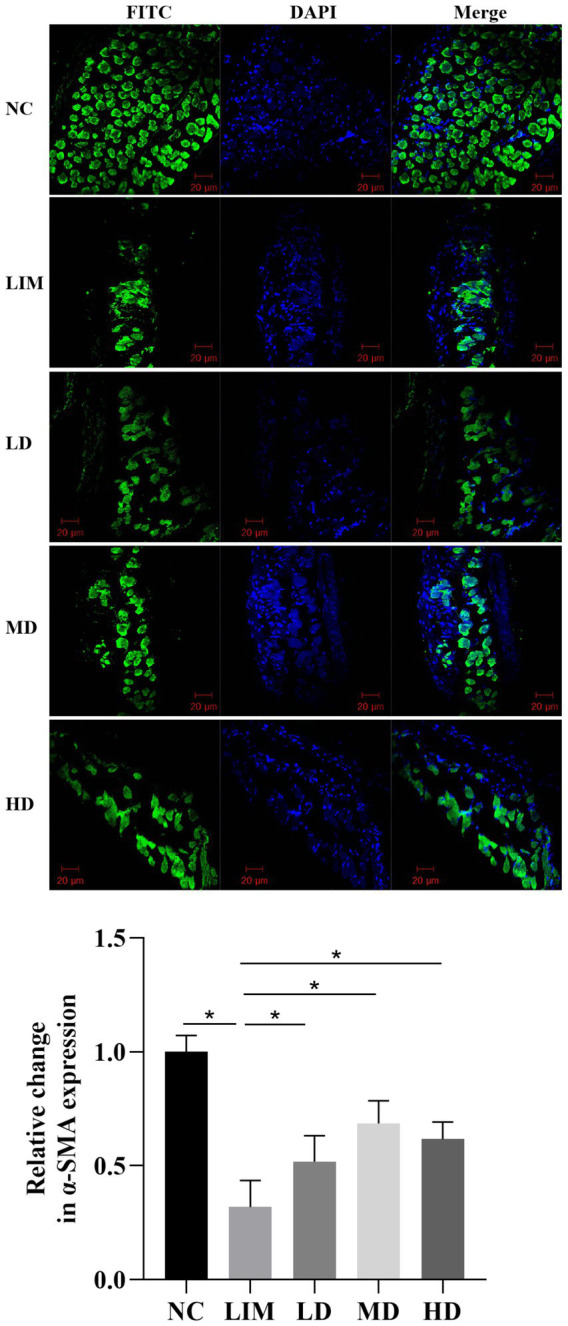
Expression and change of *α*-SMA protein in sclera observed by confocal laser microscopy. 200 times. NC, normal group; LIM, myopia group; LD, low-dose group; MD, medium-dose group; HD, high-dose group (*n* = 3) ^*^p < 0.05.

The expression of *α*-SMA protein in the sclera was observed by laser confocal microscopy using FITC labeled α-SMA protein for green fluorescence and DAPI-labeled nucleus for blue fluorescence. As shown in Figure 3, α-SMA protein expression was significantly decreased in the LIM group compared with the normal group. After intravitreal injection of rhBMP2, α-SMA protein expression in LD, MD, and HD groups was higher than that in LIM group.

### 3.5 Changes of each gene at mRNA level

**Figure 4:**
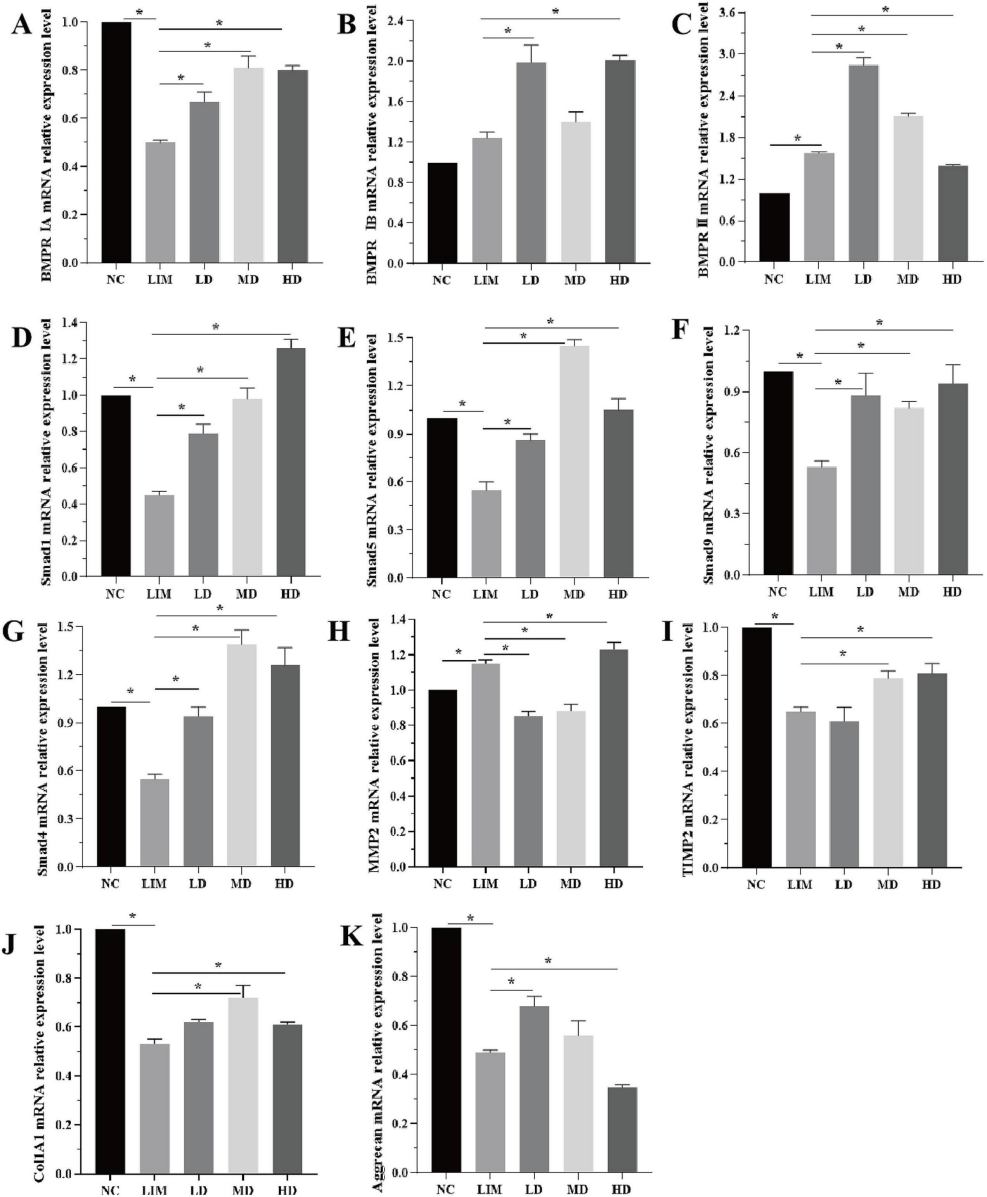
Relative expression changes of genes related to BMP2 signaling pathway at mRNA level 4 weeks after modeling **(A-K)**. ^*^p < 0.05. NC: normal group, LIM: myopia group, LD: low-dose group, MD: medium-dose group, HD: high-dose group. (*n* = 6).

As shown in Figure 4, qPCR was used to detect the changes in genes related to the BMP2 signaling pathway at the mRNA level after 4 weeks of modeling.

BMPRIA mRNA expression: Compared with the NC group, the expression of BMPRIA in the LIM group was significantly decreased (*p* < 0.05); After intravitreal injection of rhBMP2, BMPRIA expression was significantly increased in LD, MD, and HD groups compared with the LIM group (*p* < 0.05), and the increase was most significant in the MD group (Figure 4A).

BMPRIB mRNA expression: Compared with the NC group, the expression of BMPRIB in the LIM group was not significantly increased (*p* > 0.05); After intravitreal injection of rhBMP2, compared with the LIM group, BMPRIB expression was significantly increased in LD and HD groups (*p* < 0.05), but not in MD group (*p* > 0.05) (Figure 4B).

BMPRII mRNA expression: Compared with the NC group, BMPRII expression in the LIM group was significantly increased (*p* < 0.05). After intravitreal injection of rhBMP2, compared with the LIM group, the expression of BMPRII in the LD and MD groups was significantly increased (*p* < 0.05), while that in the HD group was significantly decreased (*p* < 0.05) (Figure 4C).

mRNA expressions of smad1, smad5, smad9, and smad4: Compared with the NC group, the expressions of smad1, smad5, smad9, and smad4 in the LIM group were significantly decreased (*p* < 0.05). After intravitreal injection of rhBMP2, compared with myopia group, the expressions of smad1, smad5, smad9, and smad4 in LD, MD, and HD groups were significantly increased (*p* < 0.05, Figure 4D–G).

MMP2 mRNA expression: Compared with the NC group, MMP2 expression in the LIM group was significantly increased (*p* < 0.05). After intravitreal injection of rhBMP2, compared with the LIM group, the expression of MMP2 in the LD and MD groups was significantly decreased (*p* < 0.05), while that in the HD group was significantly increased (*p* < 0.05, Figure 4H).

TIMP2 and Col1A1 mRNA expression: Compared with the NC group, the expression of TIMP2 and Col1A1 in the LIM group was significantly decreased (*p* < 0.05). After intravitreal injection of rhBMP2, compared with the myopia group, TIMP2 and Col1A1 expression was significantly increased in the MD and HD groups (*p* < 0.05), while there was no significant change in the LD group (*p* > 0.05, Figure 4I–J).

Aggrecan mRNA expression: Compared with NC group, Aggrecan expression in the LIM group was significantly decreased (*p* < 0.05); After intravitreal injection of rhBMP2, compared with the LIM group, Aggrecan expression was significantly increased in LD group (*p* < 0.05), significantly decreased in HD group (*p* < 0.05), and had no significant change in MD group (*p* > 0.05, Figure 4K).

### 3.6 Changes in protein levels of each gene

**Figure 5:**
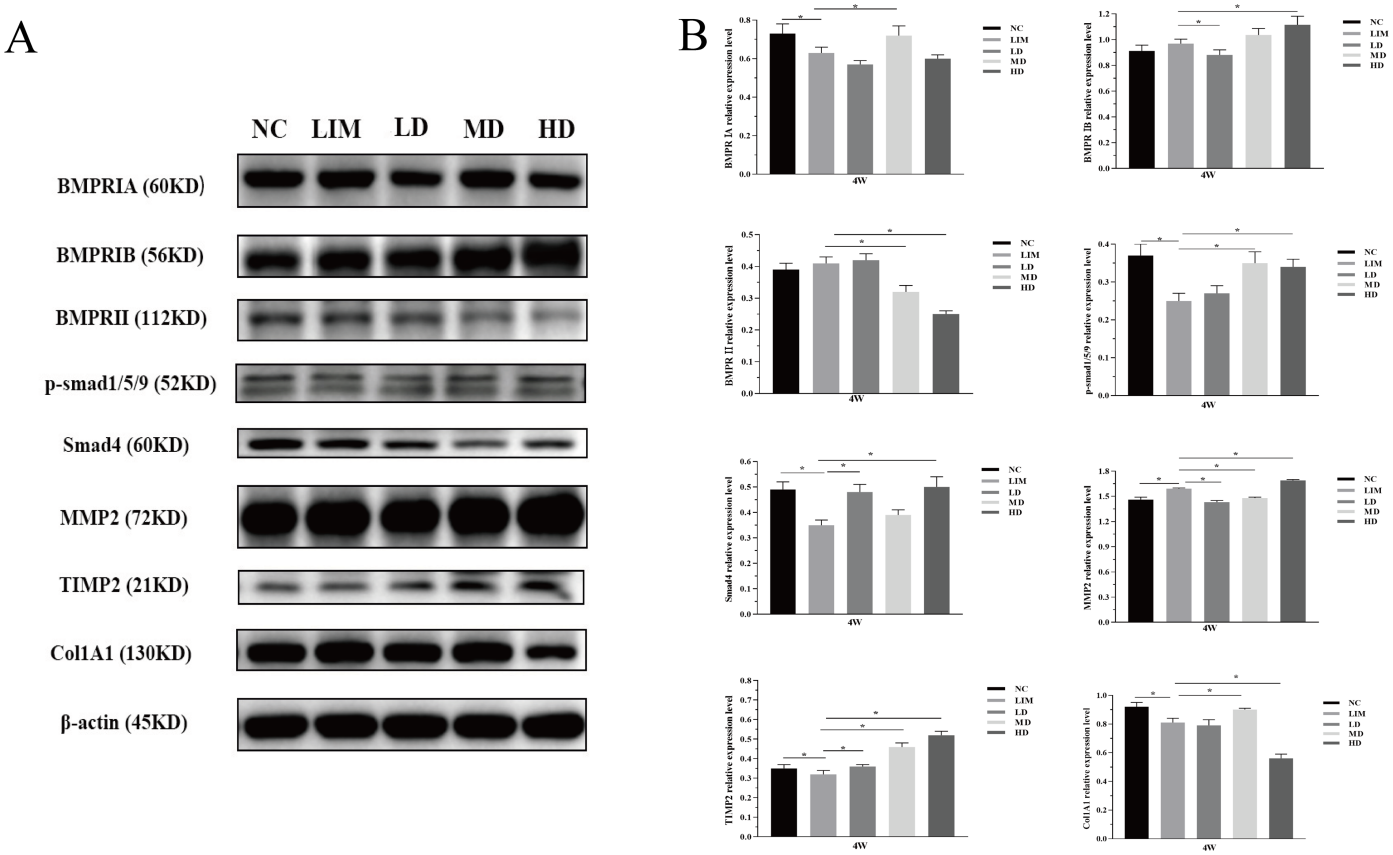
Relative expression changes of BMP2 signaling pathway related genes at protein level 4 weeks after modeling **(A,B)**. ^*^p < 0.05. NC, normal group; LIM, myopia group; LD, low-dose group; MD, medium-dose group; HD, high-dose group (*n* = 6).

As shown in Figure 5A (grayscale) and Figure 5B (bar chart), the changes of BMP2 signaling pathway-related genes at the protein level 4 weeks after modeling were detected by Western blot. The expression of BMPRIA: After 4 weeks of modeling, BMPRIA expression in the LIM group was significantly decreased compared with the NC group (*p* < 0.05). After intravitreal injection of rhBMP2, BMPRIA expression was significantly increased in the MD group, but not significantly decreased in the LD and HD groups (*p* > 0.05). BMPRIB expression: After 4 weeks of modeling, BMPRIB expression in the LIM group was not significantly increased compared with the NC group (*p* > 0.05). After intravitreal injection of rhBMP2, compared with the LIM group, BMPRIB expression was significantly decreased in the LD group (*p* < 0.05), significantly increased in the HD group (*p* < 0.05), but not significantly increased in the MD group (*p* > 0.05).

The expression of BMPR II: After 4 weeks of modeling, BMPR II expression in the LIM group was not significantly increased compared with the NC group (*p* > 0.05). After intravitreal injection of rhBMP2, BMPR II expression was significantly decreased in MD and HD groups (*p* < 0.05), but not significantly increased in LD group (*p* > 0.05) compared with the LIM group.

P-smad1/5/9 expression: After 4 weeks of modeling, compared with the NC group, the expression of p-smad1/5/9 in the LIM group was significantly decreased (*p* < 0.05); After intravitreal injection of rhBMP2, the expression of p-smad1/5/9 in the MD and HD group was significantly higher than that in the LIM group (*p* < 0.05).

Smad4 expression: After 4 weeks of modeling, Smad4 expression in the LIM group was significantly decreased compared with the NC group (*p* < 0.05); After intravitreal injection of rhBMP2, compared with the LIM group, Smad4 expression was significantly increased in LD and HD groups (*p* < 0.05), but not significantly increased in the MD group (*p* > 0.05).

MMP2 expression: After 4 weeks of modeling, MMP2 expression in the LIM group was significantly increased compared with the NC group (*p* < 0.05). After intravitreal injection of rhBMP2, compared with the LIM group, the expression of MMP2 in the LD and MD groups was significantly decreased (*p* < 0.05), while that in the HD group was significantly increased (*p* < 0.05).

TIMP2 expression: After 4 weeks of modeling, TIMP2 expression in the LIM group was significantly decreased compared with the NC group (*p* < 0.05). After intravitreal injection of rhBMP2, TIMP2 expression in LD, MD, and HD groups was significantly higher than that in the LIM group (*p* < 0.05).

Col1A1 expression: After 4 weeks of modeling, Col1A1 expression in the LIM group was significantly decreased compared with the NC group (*p* < 0.05). After intravitreal injection of rhBMP2, Col1A1 expression was significantly increased in the MD group compared with the LIM group (*p* < 0.05).

## 4 Discussion

In this study, we found that vitreous cavity injection of rBMP2 delayed the growth of myopia refraction and Axial length, and further explored the possible molecular mechanisms through the BMP2/Smad signaling pathway.

The sclera is a special connective tissue, consisting of a minimal fraction of cellular components and most of the extracellular matrix, which is located outside the eyeball and maintains the shape and structure of the eye (5, 10–12). Significant progress has been made in understanding how myopia develops through the use of animal models, with defocus lens-induced myopia models in guinea pigs being a common approach to myopia modeling, this induction is more consistent with the formation of myopia in humans. In this study, guinea pigs were induced by −6.0D lens for 4 weeks, the refraction was about −6.01 ± 0.99 D and the myopia model was successfully induced.

Previous studies have shown that BMP can activate the Smad signaling pathway specifically after binding to BMP receptors on cell membranes to form complexes. BMPR is a member of the receptor superfamily, consisting of BMPR I and BMPR II subtypes. In the classical Smad signaling pathway, type I receptors are activated, formation of ligand-receptor complexes, phosphorylation and activation of downstream transcription factors Smad1/5/9 (Smad8) proteins, and R-Smads phosphorylated at the carboxyl-terminal under the action of tyrosine kinase binding to Smad4 to form complexes, which move to the nucleus to further activate or inhibit the expression of related genes in the nucleus, thus affecting cell growth, differentiation and extracellular matrix synthesis (13, 14). In this study, 4 weeks after lens induction, at the mRNA and protein levels, the expression of BMPRIA, smad1, smad5, smad9, and smad4 was downregulated in the myopic group, and the expression of BMPRIB and BMPRII was not statistically significantly different from that of the normal group. After rhBMP2 was injected into the vitreous cavity of LIM guinea pigs, the expression of BMPRIA, smad1, smad5, smad9, and smad4 was up-regulated, which was most obvious in the middle-dose group, and the expression of BMPRIB and BMPRII mainly showed up-regulation or down-regulation and was not dose-dependent. We speculate that the concentration of rhBMP2 may affect its binding to BMPR receptor, and the effect of MD group is better than that of LD group and HD group, which may be due to the low concentration of LD group, which cannot achieve better effect. High concentrations in the HD group may lead to saturation of the target receptor, triggering negative feedback regulation (such as receptor downregulation or desensitization), thereby reducing efficacy (15), while moderate dose concentrations in the MD group may activate the receptor while avoiding overstimulation. In addition, low to moderate doses of drugs may activate adaptive protective pathways that activate animals, while high doses may overwhelm these mechanisms and even produce toxic side effects (16). rhBMP2 mainly binds to type I receptors and activates the Smad signaling pathway after binding, which further indicates that rhBMP2 can affect the occurrence and development of myopia in guinea pigs through BMP/smad signaling pathway.

Scleral fibroblasts and their extracellular matrix are key components of the sclera (17). Myofibroblasts interact with the extracellular matrix through the fibronectin linkage complex and may participate in the regulation of extracellular matrix synthesis and degradation, as well as collagen synthesis. *α*-SMA is a marker for myofibroblasts and is associated with myofibroblast contractile activity. In this study, immunofluorescence showed a significant decrease in α-SMA protein expression in 4-week LIM guinea pigs compared with the normal group. We hypothesized that reduced proliferation of scleral fibroblasts and decreased expression of α-SMA in myopic guinea pigs caused thinning of scleral tissues and weakening of tissue resistance, which in turn led to growth of the axial length, decreased tension of scleral tissues, down-regulation of fibroblast activity, and entry into a vicious circle, further accelerating the lengthening of the axial length, and promoting the development of myopia. This is consistent with previous studies (18). Intravitreal injection of rhBMP2 promoted proliferation of scleral fibroblasts, upregulation of α-SMA expression, and thickening of scleral tissue.

The key to the formation of myopia is the imbalance of collagen synthesis and degradation. In the experiments of myopia animal models, abnormal collagen production and degradation in the extra-scleral matrix was found, which resulted in the thinning of the posterior pole of the sclera, thus causing the morphological changes of the eyeball and the elongation of the axial length (19, 20). Collagen fibers in the sclera are mainly composed of type I collagen, type III collagen, and type IV collagen, of which type I collagen accounts for the vast majority and is located between the equator and the posterior pole of the eyeball. Studies have shown that the expression of collagen in myopic scleral remodeling is all decreased, and the decrease of collagen fiber expression in type I is the most significant (21, 22). Studies have shown that Col1A1 is a susceptibility gene in Chinese and Japanese populations, and hypermethylation of CpG site y in Col1A1 promoter/exon 1 May underlie reduced collagen synthesis at the myopic scleral transcriptional level (23, 24). In this study, it was found that both mRNA and protein expression levels of Col1A1 in the scleral tissues of guinea pigs in the myopia group were decreased, which was consistent with previous research results (25). Col1A1 expression was up-regulated in the medium-dose group after intravitreal injection of rhBMP2, indicating increased synthesis of type I collagen fibers.

The key to the remodeling of the extracellular matrix lies in the secretion imbalance between MMPs and TIMPs, which maintains the dynamic balance between extracellular matrix synthesis, secretion, and degradation. MMPs play a major role in the extracellular matrix-degrading enzyme family. MMP-2, the major gelatinase in animal sclera, has a significant degrading effect on a wide range of collagens, including type I collagen, and TIMP-2, an inhibitor of MMP-2, can inhibit its activity, thereby inhibiting collagen degradation (26). The imbalance of MMP-2/TIMP-2 expression may be the reason driving the change in scleral plasticity. In this study, 4 weeks after modeling, MMP2 expression increased and TIMP2 expression decreased in experimental myopic sclera at mRNA and protein levels. The above findings have also been confirmed in myopia studies (27). During the development of visual deprivation myopia, the expression level of MMP-2 in the scleral fiber layer of chickens was elevated compared with that of normal controls, while the expression level of TIMP-2 was decreased compared with that of normal controls. After intravitreal injection of rhBMP2, MMP2 expression was decreased and TIMP2 expression was increased, suggesting that rhBMP2 can inhibit scleral remodeling by regulating the expression of MMP-2/TIMP-2.

Based on the above results, we believe that after injecting rhBMP2 into the vitreous cavity of LIM guinea pigs, rhBMP2 can bind to the BMP2-related receptors, activate the Smad signaling pathway, affect the expression of MMP2/TIMP2, promote the expression of Col1A1 gene, regulate scleral remodeling, promote collagen I synthesis, and delay the development of myopia. It provides a new method for clinical treatment progress based on the molecular mechanism of myopia.

## Data Availability

The raw data supporting the conclusions of this article will be made available by the authors, without undue reservation.
